# Deciphering the archaeal communities in tree rhizosphere of the Qinghai-Tibetan plateau

**DOI:** 10.1186/s12866-020-01913-5

**Published:** 2020-08-01

**Authors:** Mengjun Zhang, Liwei Chai, Muke Huang, Weiqian Jia, Jiabao Guo, Yi Huang

**Affiliations:** State Key Joint Laboratory of Environmental Simulation and Pollution Control, College of Environmental Science and Engineering, Peking University, No.5 Yiheyuan Road Haidian District, Beijing, P.R. China 10087

**Keywords:** Rhizosphere, Archaeal community, Assembly process, Co-occurrence, Qinghai-Tibetan plateau

## Abstract

**Background:**

The Qinghai-Tibetan Plateau represents one of the most important component of the terrestrial ecosystem and a particularly vulnerable region, which harbouring complex and diverse microbiota. The knowledge about their underground microorganisms have largely been studied, but the characteristics of rhizosphere microbiota, particularly archaeal communities remains unclear.

**Results:**

High-throughput Illumina sequencing was used to investigate the rhizosphere archaeal communities of two native alpine trees (*Picea crassifolia* and *Populus szechuanica*) living on the Qinghai-Tibetan Plateau. The archaeal community structure in rhizospheres significantly differed from that in bulk soil. Thaumarchaeota was the dominant archaeal phylum in all soils tested (92.46–98.01%), while its relative abundance in rhizospheres were significantly higher than that in bulk soil. Ammonium nitrogen, soil organic matter, available phosphorus and pH were significantly correlated with the archaeal community structure, and the deterministic processes dominated the assembly of archaeal communities across all soils. In addition, the network structures of the archaeal community in the rhizosphere were less complex than they were in the bulk soil, and an unclassified archaeal group (Unclassified_k_norank) was identified as the keystone species in all archaeal networks.

**Conclusions:**

Overall, the structure, assembly and co-occurrence patterns of archaeal communities are significantly affected by the presence of roots of alpine trees living on the Qinghai-Tibetan Plateau. This study provides new insights into our understanding of archaeal communities in vulnerable ecosystems.

## Background

The rhizosphere is a narrow zone of soil that tightly surrounds growing plant roots, which secrete a variable but substantial amount of photosynthesis-derived organic carbon compounds that enable the growth and metabolic activities of soil microorganisms [1, 2]. Therefore, the rhizosphere has been considered to be one of the most complex interfaces in nature [3], where a variety of microorganisms drive multiple biogeochemical transformations including soil formation, carbon and nitrogen cycling [4, 5]. In addition, rhizosphere microbial communities also have important effects on plant growth, health, and abiotic stress tolerance [6–8]. A growing number of studies have investigated the structure and assembly process of rhizosphere microbial communities, as well as their response to the selective effects of various biotic and abiotic factors [9–11]. However, these studies largely focused on bacteria and fungi, and little is known about the structural characteristics and driving factors of archaeal communities in the rhizosphere.

In fact, archaea have been considered a substantial component of complex microbiomes [12], and have profound interactions with bacteria, fungi and viruses in a wide range of Earth’s ecosystems [13, 14]. Compared to soil bacteria and fungi, archaeal communities are usually of low abundance and have less diversity [15], and they used to be thought to occur only in extreme environments [16]. Due to the rapid development of high-throughput sequencing technology, recent studies have expanded our knowledge of the biology of the archaea and have discovered their fundamental and even crucial ecological functions including methanogenesis [17], ammonia oxidation [18], hydrocarbon degradation [19], sulfate reduction [20], etc. Thus, a better knowledge of the structure, assembly and interaction of archaeal components in soil is of great importance [12].

Several studies have investigated the diversity and composition of archaeal communities in the rhizosphere. These studies are mainly limited to rice and a few wetland plants [21, 22], as well as only focusing on a minority of archaea taxa such as ammonia-oxidizing archaea (AOA) and methanogenic archaea [23–25]. Thus, it is not very clear what the diversity and composition of the archaeal community as a whole is in the rhizosphere, especially under unique environmental stress. In addition, the rhizosphere community structure is affected by the combination of environmental variables and interactions among microbial species [26, 27]. However, given the unique cellular structure and specific metabolic pathways of archaea that enable them to survive and even thrive under various adverse environments [12, 28], several key questions about the archaeal community also need to be answered. The first question is about the assembly process of archaea in rhizosphere: is it governed by a deterministic process or stochastic process? The second question is how archaeal species interact with one another. Co-occurrence network provides new perspectives for the analysis of microbiota beyond those of simple diversity and composition [29], and can well answer the second question.

The Qinghai-Tibetan Plateau (QTP), known as the “roof of the world” and “the third pole”, is an important component of the terrestrial ecosystem, which provides many vital ecological services [30]. As one of the world’s most vulnerable habitat, the QTP region has harsh environmental conditions and is highly sensitive to environmental disturbance, which could greatly impact the distribution of organisms, especially soil microorganisms [31–33]. The knowledge about their underground microorganisms have largely been studied [34–36], but the characteristics of rhizosphere microbiota, particularly archaeal communities are inadequate. In this study, high-throughput sequencing of 16S rRNA gene amplicons was performed to exhaustively examine the archaeal communities derived from the rhizosphere of two native plants in the Qinghai-Tibetan Plateau. We aimed to investigate the effects of the rhizosphere of trees on the structure, assembly, and co-occurrence of archaeal communities in this ecologically vulnerable region. We tested the following hypotheses: 1) the archaeal community structure of tree rhizospheres are different from that of bulk soil in the QTP region; 2) the assembly of rhizosphere archaeal community are governed by deterministic processes in the QTP region; 3) the co-occurrence patterns of rhizosphere archaeal community are more complex than that of bulk soil in the QTP region.

## Results

### Soil physicochemical properties

The soil physicochemical properties significantly differed between the rhizospheres of two plant species and the bulk soil (Table 1; Table S1). The pH varied from 7.84 to 7.91, and the lowest pH was in the bulk soil. The moisture of the two plant rhizospheres were similar and were lower than that of the bulk soil. The highest content of soil organic matter (SOM) was observed in the bulk soil, and a significant difference was detected only in the rhizosphere of *P. crassifolia* compared to the bulk soil (*P* < 0.05). In addition, there were no significant differences in the content of total nitrogen (TN), alkali-hydrolysable nitrogen (AN) and total phosphorus (TP) among the two plant rhizosphere and the bulk soil, but the content of ammonium nitrogen (NH_4_^+^-N) and available phosphorus (AP) in the two rhizosphere were significantly higher than they were in the bulk soil (*P* < 0.05).

**Table 1:** Differences of soil physicochemical properties between two rhizosphere and bulk soils

Index	Bulk soil	*P. crassifolia*	P. szechuanica
pH	7.84(0.12)**a**	7.87(0.05)**a**	7.91(0.04)**a**
Moisture (%)	18.53(3.34)**a**	15.70(3.15)**a**	15.91(2.57)**a**
SOM (g/kg)	32.63(2.82)**b**	25.55(3.97)**a**	27.68(4.42)**ab**
TN (g/kg)	2.20(0.55)**a**	1.91(0.66)**a**	1.92(0.54)**a**
NH_4_^+^-N (mg/kg)	13.88(0.76)**a**	15.44(0.62)**b**	15.55(0.31)**b**
AN (mg/kg)	255.75(67.09)**a**	236.50(75.24)**a**	261.25(47.25)**a**
TP (g/kg)	0.34(0.03)**a**	0.36(0.03)**a**	0.35(0.03)**a**
AP (mg/kg)	5.50(0.27)**a**	6.03(0.33)**b**	6.03(0.28)**b**

Data are means ± SD in parentheses, and different letters in the columns indicate significant differences (Dunnett test, *P* < 0.05)

### Diversity and community composition of archaea

A total of 474,190 high-quality sequences were obtained with a median read count per sample of 39,516 (range: 30,420-54,538). The high-quality reads were clustered using > 97% sequence identity into 207 archaeal OTUs. The Good’s coverage scores (in all cases above 99.9%) and the rarefaction curves showed clear asymptotes (Fig. S1), which together indicated a near-complete sampling of the archaeal community in this study.

The diversity indices of archaeal communities varied among the rhizospheres of two plant species and the bulk soil (Table 2). The observed number of OTUs (Ob) was highest in bulk soil, followed by the rhizosphere of *P. szechuanica*, whereas the rhizosphere of *P. crassifolia* had lower numbers. Conversely, the Shannon index in the two plant rhizospheres were higher than they were in the bulk soil, and significant difference was identified only in the rhizosphere of *P. szechuanica* compared to the bulk soil (*P* < 0.05). The phylogenetic diversity (MNTD) of the two plant rhizospheres were similar, and their values were higher than that of the bulk soil.

**Table 2:** Differences in the α-diversity indices of archaeal communities between two rhizosphere and bulk soils

	Taxonomic Diversity		Phylogenetic Diversity
	Ob	Shannon	MNTD
Bulk soil	69(23)**a**	2.06(0.15)**a**	0.36(0.11)**a**
P. crassifolia	45(14)**a**	2.23(0.11)**ab**	0.43(0.22)**a**
P. szechuanica	63(14)**a**	2.34(0.03)**b**	0.44(0.06)**a**

Data are means ± SD in parentheses, and different letters in the columns indicate significant differences (Dunnett test, *P* < 0.05)

**Fig. 1:**
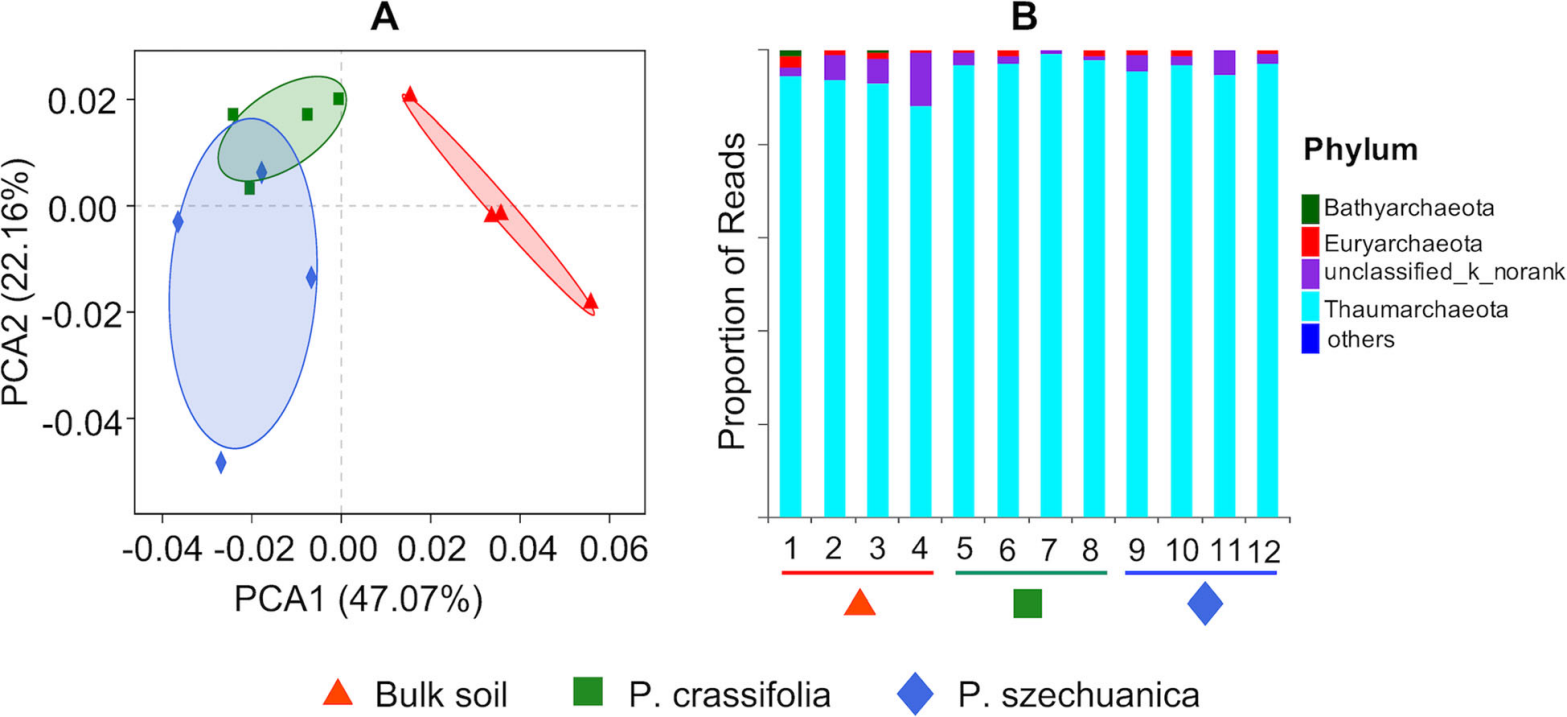
Structure and composition of archaeal communities in the two rhizosphere samples and the bulk soil. **a** Principal coordinate analysis (PCoA) ordination of archaea communities based on Weighted UniFrac distances. The 95% confidence ellipses are shown around each type of sample. **b** Taxonomic composition at the phylum level of archaeal communities

Principal coordinate analysis (PCoA) based on weighted UniFrac distances was performed to investigate the patterns of separation among archaeal microbiota. We clearly observed strong clustering of archaeal communities according to the different microhabitats (i.e., *P. crassifolia*, *P. szechuanica* rhizosphere and bulk soil). Moreover, the two plant rhizosphere samples were clearly distinguished from the bulk soil samples across the first principal coordinate, while the separation between the rhizosphere of *P. crassifolia* and *P. szechuanica* was seen along the second principal coordinate, indicating that the largest source of variation in the archaeal communities is proximity to the root, followed by plant variety (Fig. 1a). Interestingly, PCoA analysis of βMNTD distances revealed that the largest source of variation is plant variety rather than proximity to the root (Fig. S2). Consistent with the result of PCoA analyses, ANOSIM analyses also revealed significant differences in the structure of archaeal communities among the rhizosphere of two plant species and the bulk soil (Table S2).

**Fig. 2:**
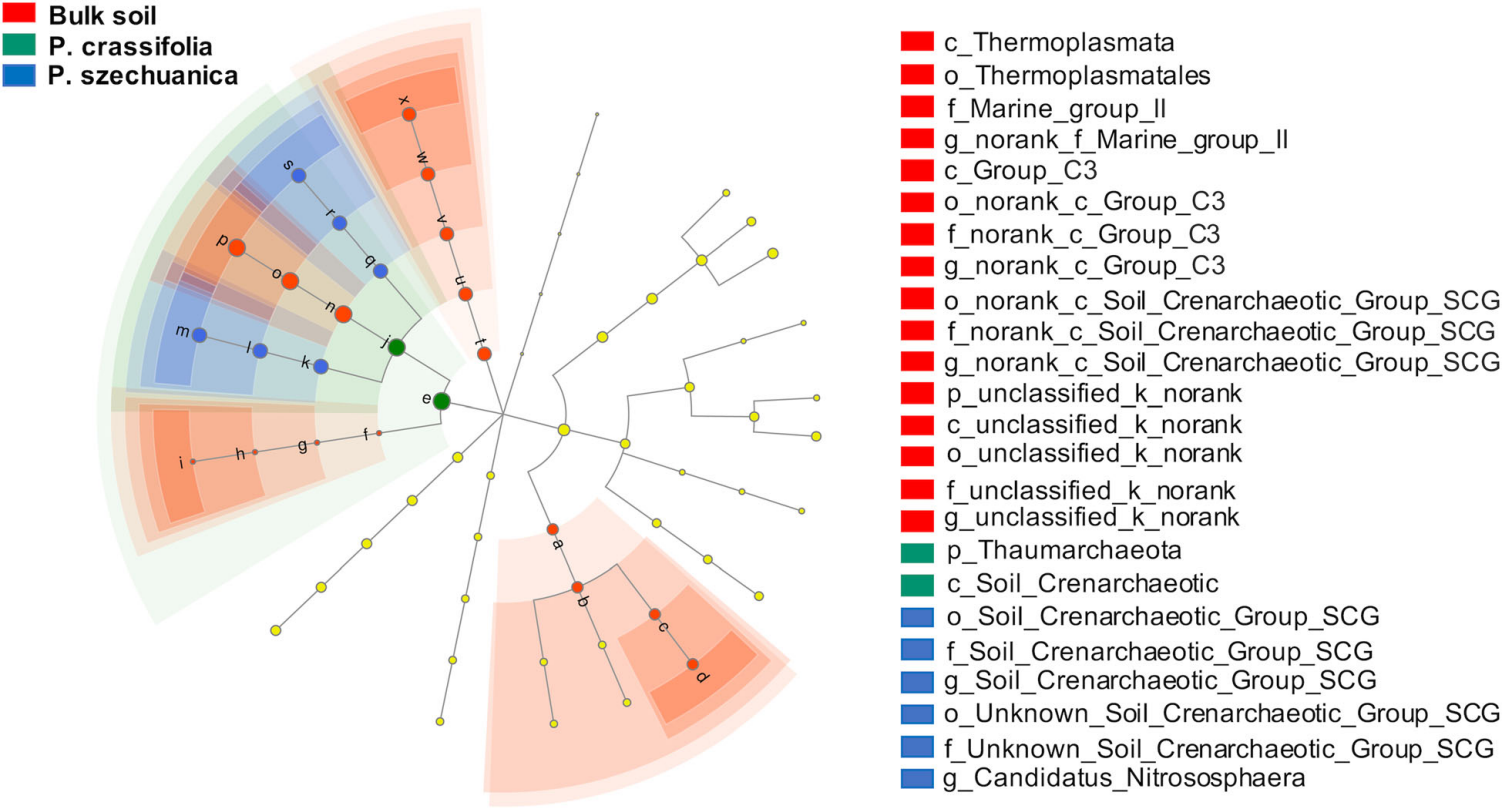
A linear discriminant analysis effect size (LEfSe) method identifies the significantly different abundant taxa of archaea. The taxa with significantly different abundances among the *P. crassifolia*, *P. szechuanica* rhizosphere and the bulk soil are represented by coloured dots. From the centre outward, they represent the kingdom, phylum, class, order, family, and genus levels. The coloured shadows represent trends of the significantly different taxa. Only taxa meeting a linear discriminant analysis (LDA) significance threshold of > 2 are shown

The relative abundance of archaeal OTUs at the phylum level was variable among the two plant rhizospheres and the bulk soil. The most dominant archaeal phyla across all samples were Thaumarchaeota, Unclassified_k_norank and Euryarchaeota, accounting for 92.46–98.01%, 1.35–6.01% and 0.56–1.18% of the pyrosequencing reads, respectively (Fig. 1b). Analysis of variance (ANOVA) showed significant enrichment of Thaumarchaeota in the rhizosphere microbiota of two plant species compared to that of the bulk soil (Dunnett test, *P* < 0.05). Conversely, the relative abundance of Unclassified_k_norank and Euryarchaeota in the rhizosphere microbiota of two plant species decreased but did not show significant differences compared with the abundance in the bulk soil (Table S3). Moreover, LEfSe analysis was also performed to determine the taxa that most likely explains the variations among different samples. In the bulk soil, four groups of archaea were significantly enriched, namely, Thermoplasmata (the class, orders of Thermoplasmatales, and its family marine_Group_II to genus), unclassified_k_norank (from phylum to genus), norank_c_Soil_Crenarchaeotic_Group_SCG (from order to genus), group_C3 (from family to genus). In the *P. crassifolia* rhizosphere, a group of archaea was significantly enriched, namely, Thaumarchaeota (the phylum and its class soil_Crenarchaeotic). In the *P. szechuanica* rhizosphere, two groups of archaea were significantly enriched, namely, unclassified_c_Soil_Crenarchaeotic_Group_SCG (from order to genus), unknown_Order_c_Soil_Crenarchaeotic_Group_SCG (from order to genus) (Fig. 2).

### Correlation between soil properties and archaeal communities

**Fig. 3:**
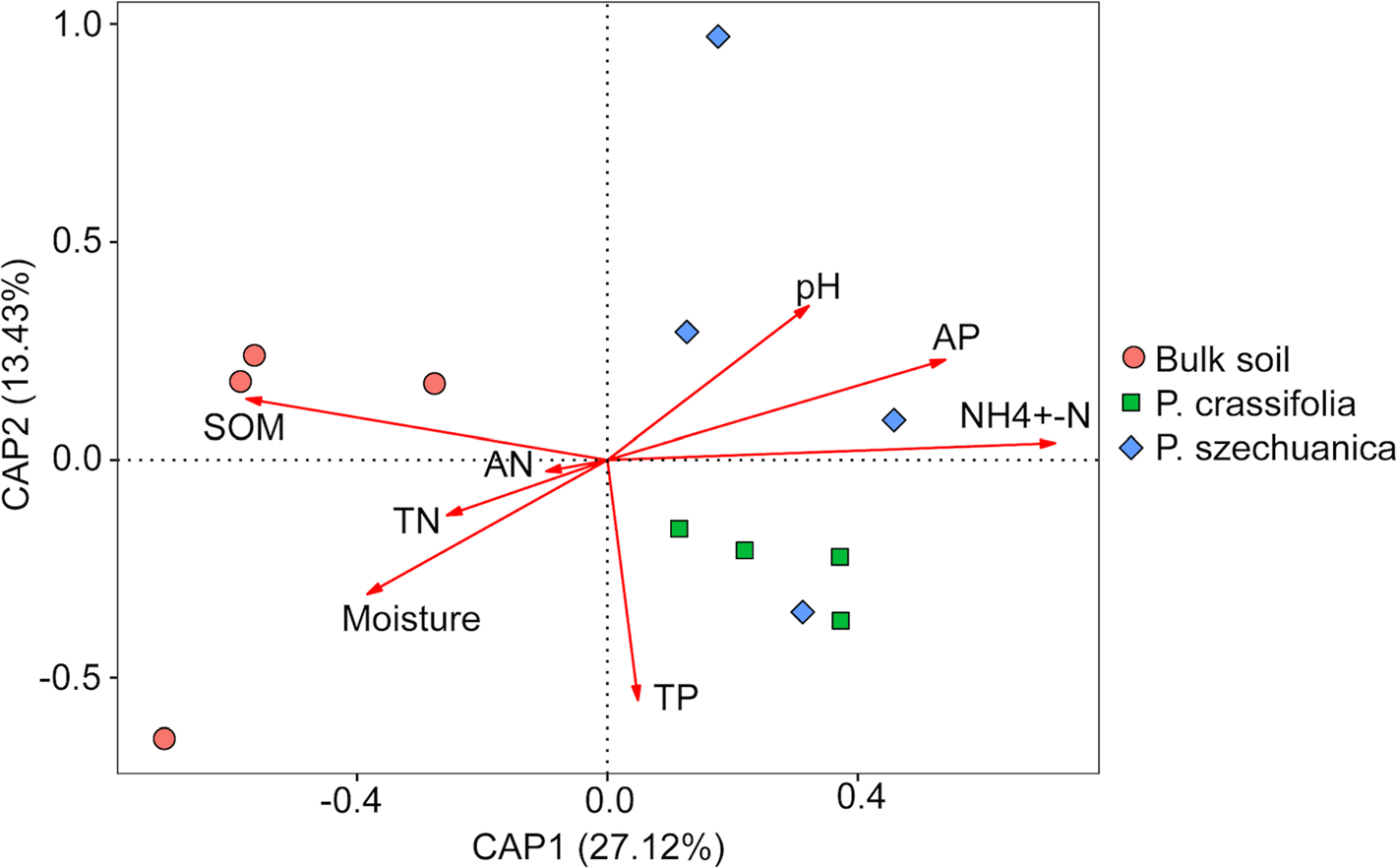
Ordination plots of the results from distance-based redundancy analysis (dbRDA) to explore the correlation between soil properties and archaeal community structure

Distance-based redundancy analysis (dbRDA) indicated the strong correlation between soil physicochemical characteristics and the structure of archaeal communities. The first two axes of CAP could explain 27.12 and 13.43% of the total variation in archaea communities, respectively (Fig. 3). In line with the PCoA (weighted UniFrac) analysis, the first axis (CAP1) could separate the rhizosphere samples from the bulk soil, and the second axis (CAP1) mainly distinguished the *P. crassifolia* rhizosphere from the *P. szechuanica* rhizosphere samples. The results of PERMANOVA analysis revealed that soil ammonium nitrogen (NH_4_^+^-N), soil organic matter (SOM) accounted for 35.1 and 28.5% of archaeal community differences, respectively, and niches (rhizosphere vs bulk soil) contributed 45.4% of the interpretation (Table S4). In addition, soil ammonium nitrogen (NH_4_^+^-N), available phosphorus (AP) and pH value were important environmental attributes significantly affecting the archaea community structure (Mantel test; *r* = 0.392, *P* = 0.026; *r* = 0.362, *P* = 0.030; *r* = 0.400, *P* = 0.028).

**Fig. 4:**
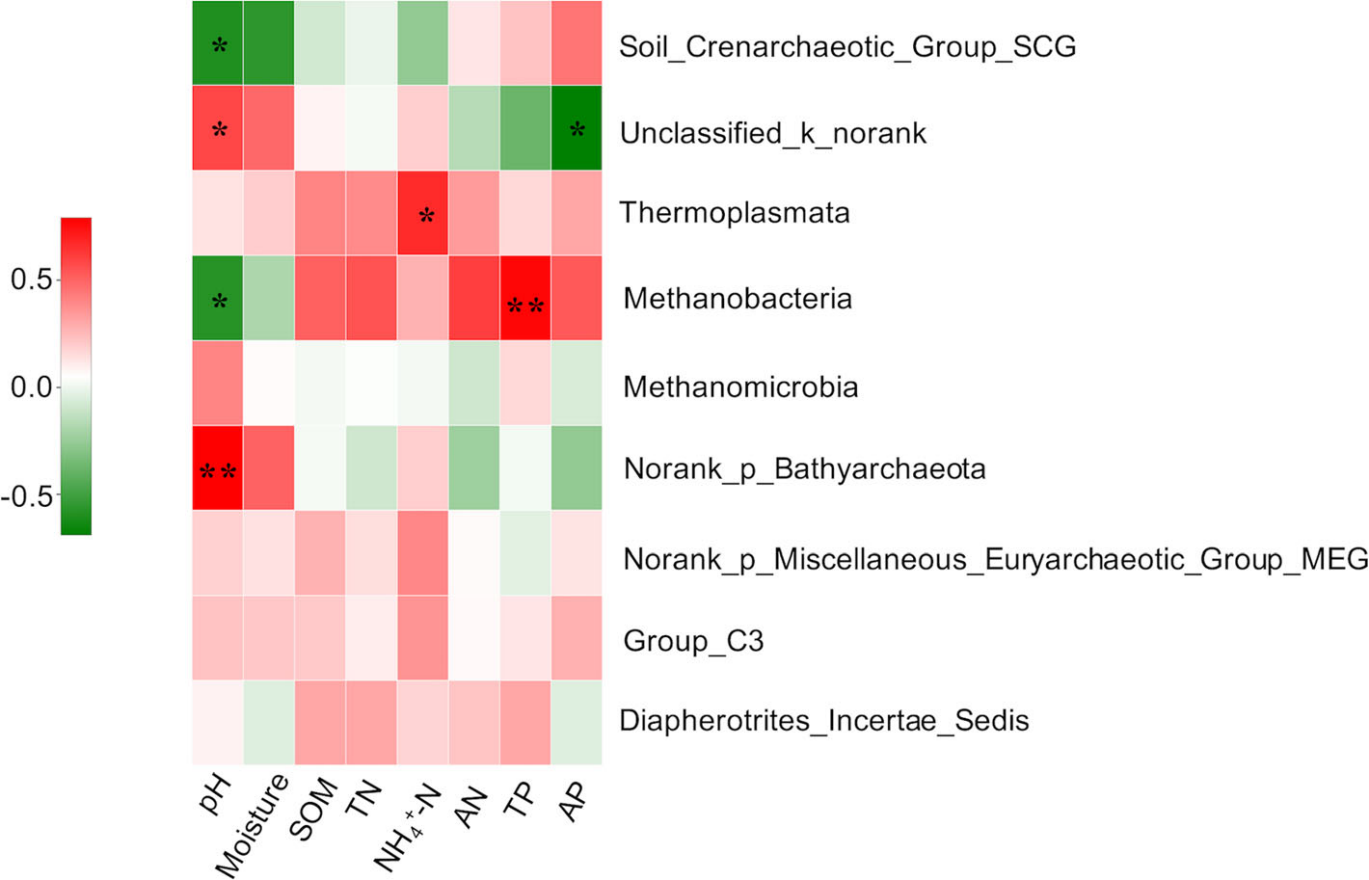
Heat map showing the Pearson correlation between soil properties and the relative abundance of the archaea taxa at the class level. The left side of the legend is the colour range of different R-values. The value of *P* < 0.05 and *P* < 0.01 is marked with “*” and “**”, respectively

Further analyses revealed that soil properties had significant effects on the relative abundance of the archaea taxa at the class level. Soil pH value was positively correlated with the relative abundance of Unclassified_k_norank, Norank_p_Bathyarchaeota, and it was negatively correlated with Soil_Crenarchaeotic_Group_SCG, Methanobacteria. Ammonium-nitrogen (NH_4_^+^-N) was positively correlated with the relative abundance of Thermoplasmata. Soil total phosphorus (TP) was positively correlated with the relative abundance of Methanobacteria. Available phosphorus (AP) was negatively correlated with Unclassified_k_norank (Fig. 4).

### Assembly processes of archaeal microbiota in rhizosphere and bulk soil

The phylogenetic tree of archaea recovered from all samples were relatively well classified according to the major lineages, and the local support values on the branches were relatively high (Fig. S3), suggesting the archaeal phylogenetic tree was reliable. Additionally, the phylogenetic signal showed that there was a significant relationship between ecological similarity and phylogenetic relatedness across short phylogenetic distances (Fig. S4). Thus, we calculated NTI and βNTI because both of these metrics emphasize phylogenetic relationships across short phylogenetic distances [37].

**Fig. 5:**
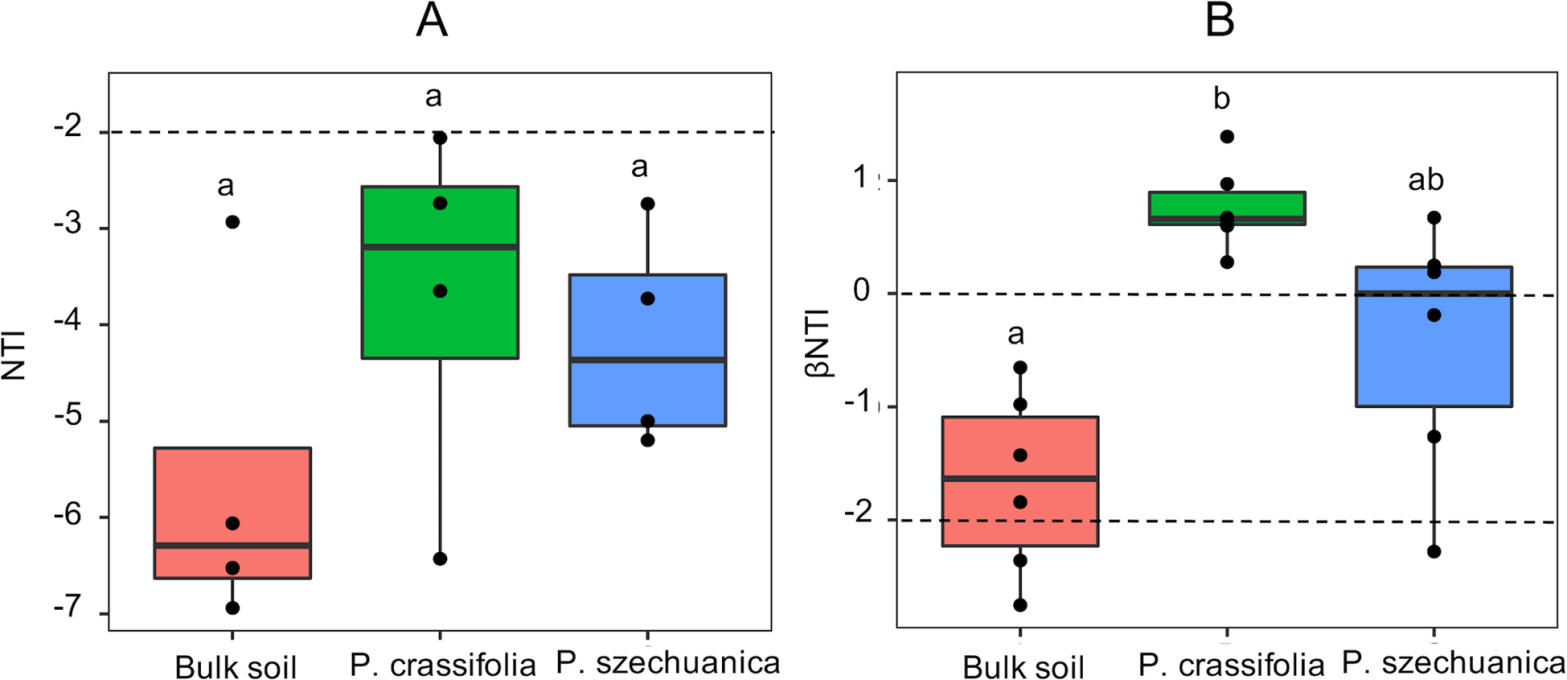
Box plot of NTI metric (**a**) and βNTI metric (**b**) of archaeal communities in all samples. Letters above the boxes indicate significant differences (Dunnett test, *P* < 0.05)

We clearly observed that the NTI values of archaea microbiota from all samples were less than − 2, in which the lowest mean NTI value was detected in the bulk soil (Fig. 5a), suggesting that archaeal communities were phylogenetically over-dispersed, especially in the bulk soil, and it also suggested that deterministic processes mainly regulate the assembly of archaeal communities. In addition, the lowest mean βNTI value for archaea was found in the bulk soil, and it was significantly lower than zero (66.67% of βNTI values ranging between 0 and − 2, 33.33% of βNTI values less than − 2), suggesting the phylogenetic turnover was less than what would be expected by chance. The mean βNTI value in the *P. crassifolia* rhizosphere was significantly higher than zero, suggesting the phylogenetic turnover was higher than what would be expected by chance. However, the mean βNTI value in the *P. szechuanica* rhizosphere was not significantly different from zero (Fig. 5b). These results further indicated that deterministic processes play a stronger role in the phylogenetic turnover than stochastic processes. Additionally, the species rank abundance distribution models also revealed that archaea communities in all samples were followed ‘niche theory’ models (Table 3).

**Table 3:** The proportion of the lowest AIC values for six species rank abundance distribution models of archaea communities in all samples

	Bulk soil	P. crassifolia	P. szechuanica
Break Stick			
Pre-emption	75%	100%	75%
Lognormal			
Zipf			
Zipf-Mandelbrot	25%		25%
ZSM			

The blank cells indicate ‘0%’

### Co-occurrence network structure of archaea microbiota

**Fig. 6:**
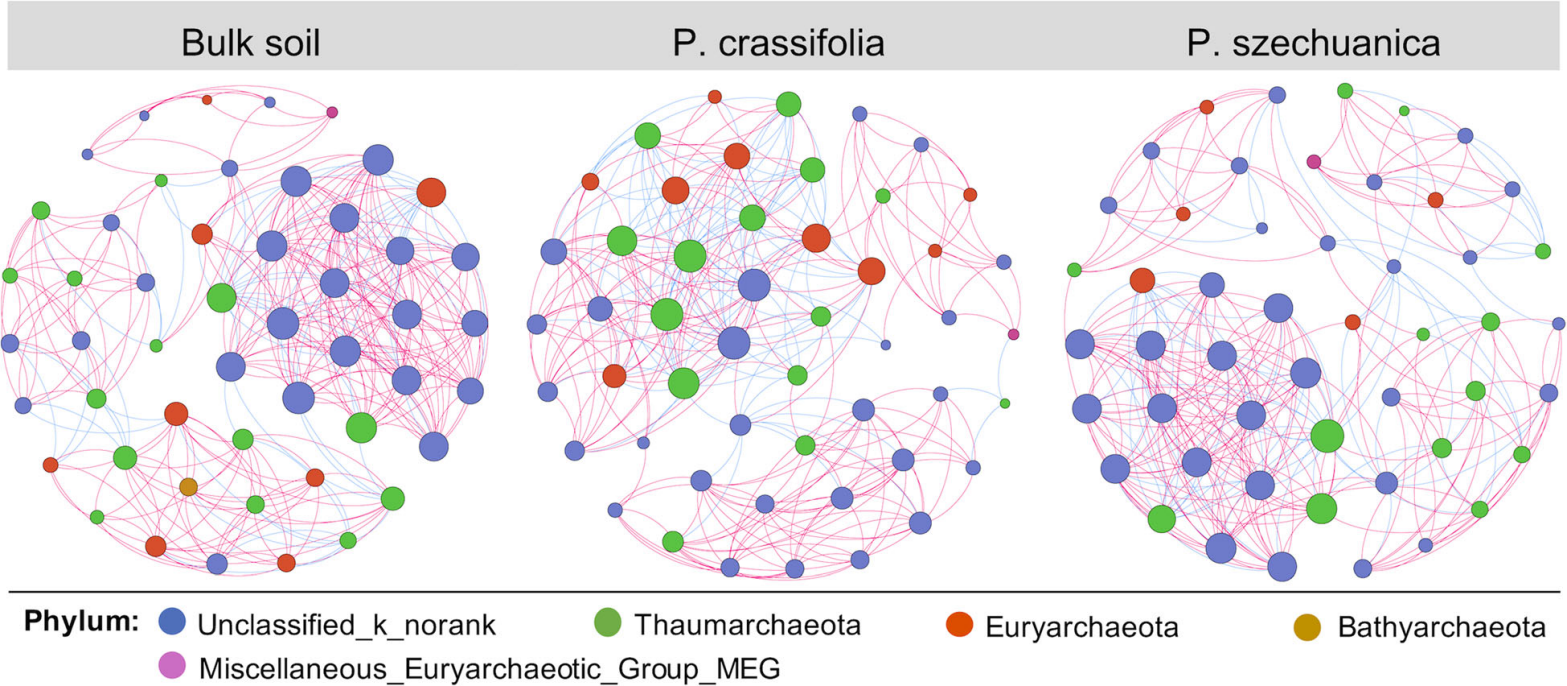
The co-occurrence network of archaea communities based on correlation analysis. Each node represents an individual OTU coloured by taxonomy at phylum level, and the size of each node is proportional to the degree. The connection stands for a strong and significant (Spearman’s |*r*| > 0.6, P < 0.05) correlation. Red edges represent positive, blue edges represent negative correlation

Three co-occurrence networks were constructed for all sample types (Bulk soil, *P. crassifolia* and *P. szechuanica* rhizosphere) to illustrate potential biotic interactions among archaea taxa (Fig. 6). All the networks were significantly different from the random networks with the identical numbers of nodes and edges (Table S5), suggesting that the network structures were non-random and reliable.

We found that the networks in the rhizosphere of two tree species were obviously different from that in the bulk soil (Fig. 6; Table S5). The number of edges, average degree and average clustering coefficient of the networks in two rhizosphere were lower than they were in bulk soil, indicating that rhizosphere assemblages of two plant species formed lower complex archaea networks compared with that of the bulk soil. The ratio of negatively correlated edges between OTUs in the *P. crassifolia* rhizosphere (30.8%) and the *P. szechuanica* rhizosphere (27.6%) were profoundly higher than that of in bulk soil (20.1%), which could be interpreted as increased competitions among archaea taxa in the rhizosphere environment. We also observed a high proportion of unclassified_k_norank in all networks (Bulk soil, *P. crassifolia* and *P. szechuanica* rhizosphere), accounting for 52.0, 52.0 and 62.0%, respectively. In addition, the majority of unclassified_k_norank were highly connected in the networks. Thus, it could be inferred that unclassified_k_norank is very crucial for the stability of archaea network structures in all samples.

## Discussion

### Variation of archaea community structures between rhizosphere and bulk soil

In this study, the structure of archaeal communities in the rhizosphere of two tree species were significantly different from that in the bulk soil in the QTP region (Fig. 1; Fig. S1; Table 2; Table S2), which supported our first hypothesis. Moreover, we found that the largest source of variation in archaeal communities is the presence of plant roots, followed by plant species, which agreed with previous studies of rhizosphere bacterial and fungal communities [38–41]. Plant roots could release a variety of carbon exudates including sugars, amino acids, organic acids, mucilage and root border cells [42, 43]. These exudates are available nutrients and energy for microbial activities [44], making the microbial community structures in the rhizosphere differed from what is found in the bulk soil [5]. The variation of archaeal communities observed between the rhizosphere *P. crassifolia* and *P. szechuanic* may be due to the difference in the composition of carbon exudates released by the roots of the two tree species [45]. In addition, the plant rhizospheres could form oxygen-depleted micro-niches for soil microorganisms due to the respiration of the roots [46]. Most of Archaea have also been identified as strictly anaerobic or facultative anaerobes [12], which are likely to be affected by the changes of redox potential in the rhizosphere. Consistent with this inference, important groups of Archaea, such as ammonium-oxidizing archaea and methanogens, have been proved to have unique distribution in the rhizosphere of *Phragmites australis* and *Halocnemum strobilaceum* [25, 47]. Therefore, this may also explain the difference of archaeal communities between the rhizosphere and bulk soil in the QTP region.

Analysis of archaeal community composition revealed that the archaeal communities were dominated by Thaumarchaeota phylum, accounting for 92.46–98.01% of sequences in this study (Fig. 1b; Table S3). This finding was in agreement with previous findings from the research also conducted in the Qinghai-Tibetan Plateau, which showed that the dominant archaeal phylum was Thaumarchaeota, accounting for 79.27% of sequences [32]. Thaumarchaeota have been detected in a variety of habitats [48–50], and identified as a novel archaeal phylum in 2008 [48]. Many studies have suggested that Thaumarchaeota species possess ammonia oxidizing abilities and are considered to play an important role in nitrogen cycling [51, 52]. In our study, the relative abundance of Thaumarchaeota in the two different plant rhizosphere were significantly higher than they were in the bulk soil (Fig. 1b; Table S3), and all the eight biomarkers in rhizosphere by the LEfSe analysis also belong to the phylum Thaumarchaeota (Fig. 2). These findings collectively indicated that the nitrogen metabolism activities occurred in the rhizosphere might be higher than that in that in the bulk soil.

### Important drivers of archaea communities

Combined with the analysis of dbRDA and Mantel test showed that soil ammonium nitrogen (NH_4_^+^-N), available phosphorus (AP) and pH were significantly correlated with the archaeal community structures (Fig. 3). This observation in NH_4_^+^-N agreed with previously reported results in the study by Norman and Barrett [53], which documented NH_4_^+^-N as a metabolic substrate that drives the distribution patterns in richness of ammonia-oxidizing archaea (AOA). Moreover, our results revealed that the content of NH_4_^+^-N in the rhizosphere was significantly higher than it was in the bulk soil (Table 1), and the increase of NH_4_^+^-N concentration in the rhizosphere may be due to the enrichment of diazotrophic bacteria in the rhizosphere, which can convert atmospheric N_2_ into ammonium via biological nitrogen fixation [54, 55], although the results of this study cannot be directly confirmed. The relative abundance of the class Thermoplasmata was positively correlated with soil NH_4_^+^-N (Fig. 4). These results corroborate the opinion that NH_4_^+^-N may play a significant role in shaping the archaeal community structures. Numerous studies have suggested that soil pH is a major driver of the community structure of bacteria, fungi or diazotrophs [56–58], but there seems to be no consensus on archaea [53, 58]. This contradiction could be explained by a plausible interpretation: soil pH indirectly affects the abundance of major archaeal taxa mainly by regulating the availability of substrates such as NH_4_^+^, CO_2_, and CH_3_COOH [53, 59, 60], so the correlation may vary with samples or by region. The significant correlation in our study could be attributed to more available NH_4_^+^-N regulated by pH to the phylum Thermoplasmata [61]. Soil available phosphorus (AP) has been reported to be a limiting factor for the growth of plants or microorganisms [7, 62]; thus, it may directly or indirectly affect archaeal communities.

### Deterministic processes govern the assembly of archaeal communities

Our results indicate that the assembly of archaeal communities were governed by the deterministic processes across all soils in the QTP region (Fig. 5; Table 3), consistent with our second hypothesis. The mean NTI values were significantly lower than zero in all samples, which provided concrete evidence that the archaeal communities were more phylogenetically over-dispersed than expected as a result of chance [63, 64]. Previous studies have shown that the competition among species would become more frequent where there was greater niche similarity and would subsequently lead to the coexistence of distantly phylogenetically related species [65, 66]. In the present study, most of the soil variables (except for NH_4_^+^-N and AP) were similar (Table 1), and microorganisms competing strongly for nutrients or water in the QTP region suffered from its low temperature and strong ultraviolet radiation [67, 68], which was supported by the high proportion of negative interactions in archaeal networks (Fig. 6; Table S5). These factors may explain why the archaeal communities were phylogenetically over-dispersed. Furthermore, the NTI values in the rhizosphere were greater than that in the bulk soil but not significant, which might indicate that stochastic processes may still play a minor role [69]. In fact, former researches have already proved that the assembly of ecological communities are regulated concurrently by both stochastic and deterministic processes [37, 70, 71]. In addition, We also found that the rhizosphere βNTI values were significantly greater than those measured in the bulk soil, suggesting that the phylogenetic turnover of archaea in the rhizosphere were higher than what was in the bulk soil [55, 71]. This could be attributed to dynamic rhizosphere microhabitats potentially stimulating the activities and evolutions of archaeal species [3].

### Distinct archaeal networks in rhizospheres and bulk soil

Previous studies have found that bacterial or fungal networks in the rhizospheres were more [40, 72] or less [27, 73] complex than what were found in the bulk soil. In the present study, we found that the archaeal co-occurrence networks of in the rhizosphere of two tree species were less complex relative to that of the bulk soil in the QTP region (Fig. 6; Table S5), which not supported our third hypothesis. Considering the complexity of microbial networks may represent ecological interactions or niche sharing among microorganisms [74], the rhizosphere of two tree species in QTP region likely fosters fewer archaeal interactions or develops less shared niches than the bulk soil, which could be explained through two plausible interpretations. On the one hand, the archaea possess distinctive metabolic pathways and enzymes that enable them to survive and thrive under extreme or nutrient-poor environments [12, 13], which may lead to their lower nutrient dependence on root exudates than bacteria or fungi. On the other hand, the rhizosphere bacterial and fungal species are likely to accelerate the consumption of substrates required by archaea, and even the plants themselves may be competitors for microorganisms under severe environmental stress in the QTP region [75, 76], thus reducing the interactions or niche sharing among archaea. This interpretation was also supported by the finding that higher numbers of negative links occurred in the rhizosphere networks. Moreover, average path length of archaeal network was smaller in the rhizospheres than in the bulk soil. Networks with small path length are considered to be small-world networks [77], which are related to the rapid responses of ecosystems to perturbations [78]. Therefore, archaeal community in the tree rhizospheres may be more sensitive to climate change compared with in the bulk soil in ecologically vulnerable region. We also found that an unclassified archaeal group, unclassified_k_norank occupies a high proportion in all networks despite its low abundance in the community composition (Fig. 1b; Table S3). Even though we do not yet know the specific ecological functions of this unclassified archaeal group, it is clear that it may play an important role in maintaining the stability of community structure and function [79].

## Conclusions

In summary, our study provides insight into the structure, assembly and co-occurrence patterns of the rhizosphere archaeal communities in the QTP region. The results showed that archaeal community structures in the rhizosphere of two plant species significantly differed from that in the bulk soil. Soil ammonium-nitrogen (NH_4_^+^-N), soil organic matter (SOM), available phosphorus (AP) and pH were important drivers of the archaeal communities. Deterministic processes dominated the assembly of archaeal communities across all samples. The network structures of the archaeal community in the rhizosphere were less complex than they were in the bulk soil. We also identified an unclassified archaeal group (unclassified_k_norank) that may be crucial for the interrelationships among archaeal species. Future research should further investigate the interaction between archaea and other microorganisms such as bacteria, fungi and protists in the rhizosphere, and work to understand the role of archaea in plant survival and growth under low-temperature stress.

## Methods

### Site and sampling

A trees field trial located in the northeast portion of the Qinghai-Tibetan Plateau (31°32′N, 92°00′E, 4531 m a. s. l), which has a plateau sub-frigid monsoon semi-arid climate with an average annual temperature of − 2.2 °C and a mean annual precipitation of 458 mm. This field trial was established in April 2010 and contains two native alpine tree species (*Picea crassifolia* and *Populus szechuanica* var. *tibetica*). These two woody plants represent the typical coniferous and broad-leaved plants living in the QTP area, respectively.

In order to ensure the representativeness of the samples, the surviving and well-growing trees (*P. crassifolia* about 2.5 m, *P. szechuanica* about 4.5 m) were selected for sample collection in July 2017. Three subsamples of fine roots (< 2 mm) were carefully collected from different positions in the rhizosphere of each selected tree at the depth of 5–15 cm below ground level. The homogeneous rhizosphere soil was obtained from the combined fine root samples of each tree according to the procedure described in a previous study [38]. The bulk soil was collected from four treeless quadrats (3 m × 3 m) at the depth of 5–15 cm below ground level. Each quadrat is about 10 m away from the sampled trees, in which five soil subsamples were obtained and combined into a representative bulk soil sample. All soil samples were handpicked to remove roots and impurities, and then divided into two subsamples. One portion was air dried and sieved through 2 mm meshes for soil property analyses, and the other portion was stored at − 80 °C for DNA extraction.

### Soil physicochemical properties analysis

Soil physicochemical properties in both rhizosphere and bulk soils were analysed according to Zhou et al. [80]. Briefly, soil moisture was quantified gravimetrically by drying fresh soils in 105 °C for 48 h. Soil pH was measured by a pH meter in a soil suspension with an air-dried soil to water radio of 1: 2.5 mass/volume. Soil organic matter (SOM) was determined by the potassium dichromate oxidation titration method. Total nitrogen (TN) was measured by the Kjeldahl digestion method. Total phosphorus (TP) was determined by the Mo-Sb anti-spectrophotometric method. Alkali-hydrolysable nitrogen (AN) was measured by the alkali-hydrolysed diffusing method. Ammonium nitrogen (NH_4_^+^-N) was measured using indophenol blue spectrophotometry. Available phosphorus (AP) was extracted with a NH_4_F/HCl solution, which was then determined using a UV-visible spectrophotometer.

### DNA extraction, PCR amplification, and sequencing

Total genomic DNA was extracted using the DNeasy PowerSoil Kit (Qiagen, Hilden, Germany) according to the manufacturer’s instructions. The quantity and quality of DNA was evaluated with a spectrophotometer (NanoDrop, ND2000, Thermo Scientific, Wilmington, DE, USA). The primers 524F10extF (5′-TGYCAGCCGCCGCGGTAA-3′) and Arch958RmodR (5′-YCCGGCGTTGAVTCCAATT-3′) were chosen for the amplification and subsequent high-throughput sequencing of the archaea [81]. Each sample was amplified in triplicate in a 20 μL mixture containing 4 μL of 5 × FastPfu Buffer, 2 μL of 2.5 mM dNTPs, 0.8 μL of each primer (5 μM), 0.4 μL of FastPfu Polymerase and 10 ng of template DNA. The PCR reaction were carried out using the following protocol: 3 min of denaturation at 95 °C, 27 cycles of 30 s at 95 °C, 30 s of annealing at 55 °C, 45 s of elongation at 72 °C, and a final extension at 72 °C for 10 min. The PCR products were extracted and purified by agarose gel electrophoresis, and further quantified using QuantiFluor™-ST (Promega, USA) according to the manufacturer’s protocol. Purified amplicons were pooled in equimolar amounts and paired-end sequencing was performed on an Illumina MiSeq platform (Illumina, San Diego, USA) according to the standard protocols described by Majorbio Bio-Pharm Technology Co. Ltd.

### Sequence processing

Raw sequences yielded from Illumina sequencing were processed using QIIME 1.9.1 [82]. Paired-end reads were joined with fastq-join, demultiplexed and quality filtered with default parameters [83]. Briefly, sequences with a quality score < 20 or with any truncated reads shorter than 50 bp were removed. Operational taxonomic units (OTUs) were clustered with 97% similarity cutoff using UPARSE 7.1 and chimeric sequences were identified and removed using UCHIME. The taxonomy of each 16S rRNA gene sequence was analysed by an RDP Classifier algorithm (http://rdp.cme.msu.edu/) against the Silva (SSU123) 16S rRNA database using a confidence threshold of 80%.

### Data analysis

All statistical analyses were carried out using R (v 3.5.1, The R Core Team, 2018) unless stated otherwise. α-diversity in each sample was calculated as the observed number of OTUs (Ob), the Shannon diversity and the phylogenetic diversity (MNTD) indices. Significant differences in the variance of α-diversity and microbial abundance data were examined using one-way analysis of variance, and post hoc comparisons were conducted by the Dunnett test at the 5% level. The differences in archaeal community composition based on Weighted UniFrac and βMNTD distances were illustrated with PCoA ordination plots using the ‘cmdscale’ function from the vegan package. To statistically support the archaeal clustering patterns resulted from PCoA analysis, different samples were compared by ANOSIM analysis using the vegan package. Additionally, we performed linear discriminant analysis (LDA) coupled with effect size measurements (LEfSe) analysis to investigate statistically representative biomarkers between different samples.

Distance-based redundancy analysis (dbRDA) was carried out using the ‘rda’ function from the vegan package to explore the relationships between soil physicochemical properties and archaeal community composition. Furthermore, associations between soil properties and nine archaeal classes were evaluated by Pearson correlation analysis at the 5% level.

To evaluate the assembly processes of the archaeal community, the phylogenetic signal of each sample was first tested by following the procedure described by a previous study [69]. Briefly, environmental optima for each OTU with respect to all physicochemical variables were calculated. The correlation coefficients between phylogenetic distances and differences in environmental optima were calculated by phylogenetic Mantel correlograms [55], and the significance of these correlations were examined with 999 randomizations using the ‘mantel.correlog’ function from the vegan package. The phylogenetic diversity within each sample was calculated as the mean nearest taxon distance (MNTD) and nearest taxon index (NTI) using the ‘mntd’ and ‘ses.mntd’ functions from the picante package [84] Note that MNTD refers to the phylogenetic distance between each OTU and its closest relative also found per sample, and NTI measures the deviation of observed MNTD from MNTD in a null model with 999 randomizations. For NTI > + 2 (NTI < − 2) in a single community or a mean NTI > 0 (NTI < 0) significantly across all communities indicates coexisting taxa are more closely related (phylogenetic clustering) or more distantly related (phylogenetic over-dispersion) than can be expected by chance [37]. The pairwise phylogenetic turnover between communities was calculated as βMNTD and βNTI using the ‘comdistnt’ function from the vegan package [84]. βNTI > + 2 (βNTI < − 2) between one pair of communities or mean βNTI > 0 (βNTI < 0) significantly in all pairs of communities indicates greater (or less) than expected phylogenetic turnover, respectively [69]. If the observed βMNTD values does not significantly deviate from the null βMNTD distribution [85], it suggests that stochastic processes predominate phylogenetic community composition. In addition, to verify the results from phylogenetic analyses, five models representing niche theory (Break Stick, Pre-emption, Lognormal, Zipf, Zipf-Mandelbrot) and ZSM representing neutral theory were performed using the function ‘radfit’ from the R package ‘vegan’ or TeTame [64]. Akaike Information Criterion (AIC) was used to evaluate the fitting quality of each statistical model, where the lower AIC value indicated a better fit for the model [86].

Network analyses based on Spearman’s rank analysis were carried out with the ‘WGCNA’ package [30, 87], and structural attributes of the overall networks including average degree, clustering coefficient and average path distance were calculated in the ‘igraph’ package. The 50 most abundant OTUs of the archaea community in each sample were selected, and the co-occurrence patterns of archaea communities were explored based on strong and significant correlation (Spearman’s |*r*| > 0.6, *P* < 0.05). Finally, the constructed networks were visualized using Gephi 0.9.2 [88].

## Supplementary information

**Additional file 1 Table S1.** Soil physicochemical properties of the rhizosphere and bulk soils. Data are means ± SD in parentheses, and different letters in the columns indicate significant differences (*P* < 0.05). **Table S2.** ANOSIM analyses of separable compartments on archaeal community beta diversity distance matrix. P. crassifoli and P. szechuanica means the rhizosphere soil of *Picea crassifolia* and *Populus szechuanica*. **Table S3.** Comparison of sample differences in abundance of phyla. Data are means ± SD in parentheses. Different letters indicate significant levels (Dunnett test, *P* < 0.05). For abbreviations, see **Table S1**. **Table S4** Factors affecting the structure of archaeal communities in the rhizosphere and the bulk soil revealed by PERMANOVA. NS means not significant. **Table S5.** Co-occurrence network topological features statistics in three compartments. For abbreviations, see **Table S1**. **Figure S1.** Rarefaction curves comparing the number of sequences with the number of observed OTUs for archaeal communities in each sample. For abbreviations, see Table 1. **Figure S2.** Principal coordinate analysis (PCoA) ordination of archaeal communities based on MNTD index. For abbreviations, see Table 1. **Figure S3.** The phylogenetic trees of archaea. The numbers above each split were local support values. **Figure S4**. Mantel correlogram between the phylogenetic distances of pairwise OTUs and their niche distance of archaea.

## Data Availability

The raw archaeal sequences in this study were deposited in Sequence Read Archive (SRA) of NCBI database and were available under accession number SRP193081.

## Abbreviations

PCR: Polymerase chain reaction
rRNA: Ribosomal RNA
OTU: Operational taxonomic units
Ob: The observed number of operational taxonomic units
MNTD: Mean Nearest phylogenetic taxon distance
NTI: Nearest taxon index
PCoA: Principal coordinate analysis
dbRDA: Distance-based redundancy analysis
ANOVA: One-way analysis of variance
ANOSIM: An analysis of similarities
PERMANOVA: Permutational multivariate analysis of variance
LEfSe: Linear discriminant analysis effect size
LDA: Linear discriminant analysis
AOA: Ammonia-oxidizing archaea
SOM: Soil organic matter
TN: Total nitrogen
AN: Alkali-hydrolysable Nitrogen
NH_4_^+^-N: Ammonium nitrogen
TP: Total phosphorus
AP: Available phosphorus
