# Supplementary figures and images for: Deciphering the archaeal communities in tree rhizosphere of the Qinghai-Tibetan plateau
Source: BMC Microbiol. 2020 Aug 1;20:235. doi: 10.1186/s12866-020-01913-5 (PMC7395985; doi:10.1186/s12866-020-01913-5)

Phylogenetic tree

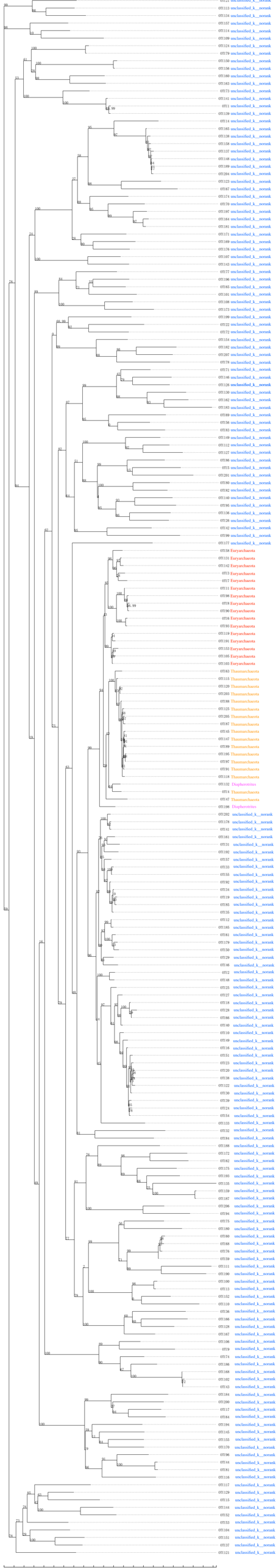

Supplement: Supplementary file 1 — Additional file 1 Table S1. Soil physicochemical properties of the rhizosphere and bulk soils. Data are means ± SD in parentheses, and different letters in the columns indicate significant differences (P < 0.05). Table S2. ANOSIM analyses of separable compartments on archaeal community beta diversity distance matrix. P. crassifoli and P. szechuanica means the rhizosphere soil of Picea crassifolia and Populus szechuanica. Table S3. Comparison of sample differences in abundance of phyla. Data are means ± SD in parentheses. Different letters indicate significant levels (Dunnett test, P < 0.05). For abbreviations, see Table S1. Table S4 Factors affecting the structure of archaeal communities in the rhizosphere and the bulk soil revealed by PERMANOVA. NS means not significant. Table S5. Co-occurrence network topological features statistics in three compartments. For abbreviations, see Table S1. Figure S1. Rarefaction curves comparing the number of sequences with the number of observed OTUs for archaeal communities in each sample. For abbreviations, see Table 1. Figure S2. Principal coordinate analysis (PCoA) ordination of archaeal communities based on MNTD index. For abbreviations, see Table 1. Figure S3. The phylogenetic trees of archaea. The numbers above each split were local support values. Figure S4. Mantel correlogram between the phylogenetic distances of pairwise OTUs and their niche distance of archaea. [file 12866_2020_1913_MOESM1_ESM.zip › Fig S3.pdf]
